# Mental Models of Illness during the Early Months of the COVID-19 Pandemic

**DOI:** 10.3390/ijerph19116894

**Published:** 2022-06-04

**Authors:** Mary Grace Harris, Emma Wood, Florencia K. Anggoro

**Affiliations:** College of the Holy Cross, Worcester, MA 01610, USA; emwood21@g.holycross.edu (E.W.); fanggoro@holycross.edu (F.K.A.)

**Keywords:** mental models, COVID-19, illness concepts, causal reasoning

## Abstract

The COVID-19 pandemic and its profound global effects may be changing the way we think about illness. In summer 2020, 120 American adults were asked to diagnose symptoms of COVID-19, a cold, and cancer, and to answer questions related to the diagnosis, treatment, prevention, time-course, and transmission of each disease. Results showed that participants were more likely to correctly diagnose COVID-19 (91% accuracy) compared to a cold (58% accuracy) or cancer (52% accuracy). We also found that 7% of participants misdiagnosed cold symptoms as COVID-19, and, interestingly, over twice as many participants (16%) misdiagnosed symptoms of cancer as COVID-19. Our findings suggest a distinct mental model for COVID-19 compared to other illnesses. Further, the prevalence of COVID-19 in everyday discourse—especially early in the pandemic—may lead to biased responding, similar to errors in medical diagnosis that result from physicians’ expertise. We also discuss how the focus of public-health messaging on prevention of COVID-19 might contribute to participants’ mental models.

## 1. Introduction

As of spring 2022, the world has been grappling with the COVID-19 pandemic for over two years. In many ways, the pandemic feels like old news; “pandemic fatigue” leads to many individuals relaxing their safety precautions both in and outside of the home [1]. Despite these feelings of exhaustion, however, the virus continues to evolve—the Omicron variant recently took over the United States with case counts skyrocketing past previous record highs, demonstrating the variant’s increased transmissibility and immune evasion [2]. Though individuals may consider themselves weathered experts in COVID-19, having endured the pandemic for so long, the fact remains that COVID-19 is an unprecedented infectious disease, especially as new variants present new challenges, and much of society has had to continuously update their beliefs about the virus.

Indeed, many basic assumptions about the spread and prevention of COVID-19 have changed drastically since the beginning of the pandemic—a time of heightened uncertainty. Some individuals armed themselves with gloves and masks and disinfected their groceries, while others held fast to the idea that COVID-19 was simply a bad case of the flu [3]. Even the science was confusing early on; some reports said the virus could be spread through airborne droplets, while others emphasized cleaning surfaces and hands in attempts to avoid fomite-based transmission (i.e., [4,5]). Many individuals did their own “research”, leading some to science-based conclusions (that were, notably, highly subject to change as information emerged) and others to conspiracy theories [6].

Clearly, the perceptions and knowledge that motivate health behaviors in the public are not always consistent with science, which is problematic because public-health strategy in combating COVID-19 has focused on creating behavioral changes in the public [7]. It is imperative to assess individuals’ mental models, or their understanding of concepts and operations related to how these concepts work, and identify potential inaccuracies regarding COVID-19 so that health education can address them more specifically [8]. Having a more accurate understanding of COVID-19 may encourage the behavioral changes necessary to prevent its spread. Individuals may compare COVID-19 to pre-existing viruses in an attempt to contextualize the new, mysterious disease in terms of illnesses about which we have decades of information and knowledge. This may pose obstacles in acquiring an accurate, science-informed mental model, as beliefs about other illnesses do not always apply to facts about this novel coronavirus [7].

Furthermore, in a study which assessed precautionary behavior related to COVID-19 in three severely impacted cities in Saudi Arabia, researchers noted that risk perception played a large role in the behavior of adults. Participants were concerned about their community, for example, economic impacts, and were weary of attending medical spaces and of medical workers in general despite believing in following precautionary guidelines [9]. New research on the impact of COVID-19 continues to be produced at a rapid rate, but few studies focus on how the COVID-19 pandemic is shaping not only mental models of coronaviruses, but other illnesses in general. As such, we were interested in whether the well-documented tendency of U.S. participants to reference germs and other biological reasons in their explanations of how individuals get sick (e.g., [10]) would be influenced by the deluge of environmental and behavioral guidelines given in media and news sources during the COVID-19 pandemic.

Prior research demonstrates a correlation between how individuals think about diseases and the preventative measures in which they partake [11]. When asked what other diseases they thought about when considering COVID-19, participants (18+) who associated COVID-19 with either colds or the flu were less likely to report taking preventative measures, such as handwashing or avoiding crowded and/or public spaces [11]. Individuals who associated COVID-19 with more severe or worrisome illnesses (i.e., pneumonia, Ebola) were more likely to report following Centers for Disease Control (CDC) recommended behaviors (excluding wearing a mask, as at the time of their data collection face coverings were not yet suggested) [11]. It is imperative to understand how people think and what they think they know about COVID-19, as these beliefs guide their personal health behaviors, may impact public health in general, and, ultimately, the course of the pandemic.

The goal of this study, conducted early in the pandemic, was to assess participants’ understanding of COVID-19. While previous research has addressed risk perception and protective health behaviors (i.e., [9,11]), no studies have attempted to examine how COVID-19 interacts with participants’ reasoning about basic illness concepts. Thus, the current study asked two questions: (1) how adults conceptualize the novel illness COVID-19, and (2) whether and how the constantly evolving discourse surrounding the COVID-19 pandemic is reflected in adults’ mental models of illness.

To address these questions, we conducted an online survey with adults of various ages living in the U.S. The surveys were administered in July 2020, when U.S. cases were increasing steadily (a week before data collection began the 7-day average number of new cases had increased nearly two-fold to 65,633 [12]). Participants were presented with a series of vignettes describing a character who exhibited symptoms of either COVID-19, the common cold, or cancer (as a general disease). After reading each vignette, participants were asked to diagnose the illness. We then asked participants to answer questions about the causes, transmission, time-course, prevention, and treatment of the illness they had just diagnosed.

We focused especially on how COVID-19 is conceived in relation to other illnesses, namely the common cold (an illness often compared to COVID-19) and cancer (an illness rarely compared with COVID-19) by asking participants to diagnose and respond to questions involving the three diseases. Pandemic-related discourse, particularly at the onset of the pandemic, regularly made explicit comparisons between COVID-19 and the flu; such a connection was not drawn between COVID-19 and colds, though colds and the flu are often spoken of in tandem (i.e., “cold and flu season”) and have a great deal of overlap in symptomatology. As such, involving colds, the less-frequently-referenced disease, was a subtler way to see how the discourse around COVID-19 may or may not relate to how we discuss more normalized illnesses. Cancer, on the other hand, is not often compared to COVID-19. The two illnesses differ in many more ways than COVID-19 and colds—for example, a much longer onset of disease, varied symptoms, and being non-contagious separates cancer from virus-caused everyday illnesses [13,14]. Thus, cancer was included as a comparison point and allowed us to observe mental models for illness across a wider range of diseases. Findings from this research can provide us with a better understanding of how individuals thought about COVID-19 early in the pandemic, information which is valuable as it pertains to health behavior and illness-related science understanding on a larger scale.

## 2. Method

### 2.1. Participants

We recruited 122 participants (72% female; 98% white) through social media posts (e.g., Instagram, Facebook, and Snapchat). Posts on Instagram and Snapchat were received mainly by college-aged students and Facebook messages were posted to community pages for the researchers’ hometowns, reaching a wider range of adults. There was no explicit mention of COVID-19 in the messages.

Participants represented a wide age range, though the majority were aged 55–64 (36.1%) or 18–24 (27.1%). The remaining participants were aged 45–54 (16.4%), 25–34 (9%), 65 or older (7.4%), or 35–44 (4.1%). Most participants were well educated, with the vast majority having at least some college education (97.5%) and many having a master’s degree or higher (37.7%). Nearly half of participants reported being Catholic (42.6%) while a high number of others claimed to be spiritual, but not religious (18%). Over half of the participants reported having no medical experience (52.5%).

### 2.2. Materials and Procedure

Participants’ consent and demographic information were collected in Google Forms. Participants were then linked to a Qualtrics survey composed of vignettes and questions that asked about their conceptions of COVID-19, cold, and cancer. The vignettes presented were adapted from Legare and Gelman [15] and McCann and Anggoro [16] to include symptoms of cancer, colds, and COVID-19 without referring to any of the illnesses by name. The goal of the vignettes was to provide sufficient information about the illness without influencing participants’ reasoning about how it was contracted. Subsequent questions asked about the cause, transmission, time course, prevention, and treatment of COVID-19 and other illnesses. Below is the vignette illustrating COVID-19 symptoms (see Appendix A for cancer and cold vignettes).

*Bill is a 35-year-old man who is seeing his doctor because of a fever, nausea, and a persistent cough. Two days ago, he started feeling fatigue and was having headaches off and on. These symptoms have progressively gotten worse. Bill also reports that he feels congested and has lost his sense of taste and smell. He is becoming more concerned about his symptoms after he started having trouble breathing earlier this morning and is now feeling some pressure on his chest.*

In addition to Bill, the vignettes included Julia, who had symptoms of cancer, and Max, who had symptoms of a cold. Reference to these specific names may be made when participant responses deal with the actions of the characters in the vignettes. Participants received the vignettes in random order. After each vignette they were asked the following questions:

- What do you think (X) is suffering from? (*Diagnosis*)
- Why/how do you think (X) got sick? Are there any other reasons? (*Cause*)
- Can other people contract what (X) is suffering from? If so, how are they most likely to contract (X)’s condition? (*Transmission*)
- After they first contract the condition, how long do you think it would take for someone to feel symptoms? (*Time Course*)
- What could (X) have done to stop himself from getting this condition? (*Prevention*)
- What, if anything, would make (X) feel better? Is there a cure for someone with this condition? (*Treatment*)

All the questions included in the survey were open-ended, allowing participants to be as concise or detailed as they wanted. A coding scheme was constructed to parse responses into meaningful units. The coding scheme was different for each question, as each was aimed at a different dimension of illness. For questions related to cause, transmission, and prevention, the coding scheme had categories related to biological agents, biological vulnerability, environmental factors, a combination of the other categories, or “other”. The time course question had shorter-term categories (i.e., 0–24 h, 1–7 days), mid-term categories (especially related to COVID-19 incubation periods, i.e., up to 2 weeks) and long-term categories (months, over a year). The treatment question included categories related to biomedical and/or self-provided treatment options. The full coding scheme is in Appendix B.

The study consisted of the two online surveys. The Google Form was distributed first and collected informed consent and asked demographic questions. The Qualtrics survey followed and was composed of four blocks. The first three blocks were given in random order and consisted of the COVID-19, cancer, and cold vignettes. Each vignette was followed by six short-response questions regarding different mechanisms of illness. The order of the questions within each block remained the same in all conditions. The last block included questions regarding the similarity between the three illnesses, as well as questions about how participants’ lives have or have not been impacted by the COVID-19 pandemic.

## 3. Results

### 3.1. Diagnosis Accuracy

We first examined participants’ ability to accurately diagnose the intended illness across the three vignettes. A response was considered “correct” if it identified the intended illness described by the vignette. Anything other than the intended illness, such as responding “strep throat” for the cold vignette, was coded as incorrect. The complete data set provided by all 122 participants showed that participants were more likely to correctly diagnose COVID-19 (93% accuracy) compared to a cold (60% accuracy) or cancer (51% accuracy), χ^2^ = 45.52, *p* < 0.00001. A similar pattern held when we included only complete responses (*n* = 67), with COVID-19 having the highest accuracy (91%), the cold the second highest accuracy (58%), and cancer least accurate (52%).

### 3.2. COVID-19 Overextensions

A closer analysis of participants’ misdiagnoses revealed an interesting finding. Of participants who misdiagnosed the common cold, 7.1% (*n* = 2) misidentified it as COVID-19. However, twice as many participants (15.6%) (*n* = 5) misdiagnosed cancer and called it COVID-19 (this included only participants who also provided diagnoses for all three vignettes [enabling full comparison] and responded to at least three of the questions for each vignette). All participants who misdiagnosed a disease as COVID-19 when it was not the intended illness correctly identified COVID-19 in its vignette. Though these numbers are small, they are notable: the symptoms included in the COVID-19 vignette and cancer vignette shared almost no overlap, suggesting participants include a vast array of symptoms in their mental models for COVID-19. Further analysis showed that three of the participants who misdiagnosed cancer as COVID-19 provided a non-COVID-19 (but nonetheless inaccurate) response to the cold vignette (i.e., “allergies”). Two participants were correct in both the COVID-19 and cold vignettes but diagnosed the cancer vignette as COVID. Of the participants who misdiagnosed the cold as COVID-19, one diagnosed cancer correctly and the other did not (calling it Lyme disease instead). No participant gave a COVID-19 diagnosis for all three vignettes.

It is notable that participants mistook COVID-19 for illnesses that were both less severe (colds) and more severe (cancer). Comparison of participants’ responses for individual illness dimensions within their two diagnoses (COVID-19 actual and their misdiagnosis) may provide more insight into how exactly these misdiagnoses occurred.

### 3.3. Participants Who Misdiagnosed the Cold as COVID-19

The first participant who misdiagnosed the character in the cold vignette as having COVID-19 provided similar answers to the actual COVID-19 vignette as for the cold vignette. There were slight differences; though the participant suggested for both vignettes that the disease was caused by exposure to another person with the virus, they simply stated that the illness in the actual COVID-19 vignette was “definitely” contagious, without elaborating as to how. In the COVID-overextended (actual cold) vignette, the participant suggested that the virus could be spread through “personal contact” and suggested “personal distancing” for both vignettes. The second participant who identified the illness in both the COVID-19 and cold vignettes as COVID-19 was generally consistent in their responses to the two vignettes (this participant diagnosed the cold vignette as either COVID-19 or the flu—only data relating to COVID-19 [when the participant made the explicit distinction] are included above). However, this participant provided a shorter time course (1–7 days) than the previous one (7–14 days), evidence of some of the aforementioned lack of consensus in participants’ apparent understanding of COVID-19.

### 3.4. Participants Who Misdiagnosed Cancer as COVID-19

Interestingly, five participants (15.63%) overextended their COVID-19 diagnosis to the cancer vignette. The cancer vignette included few symptoms that overlapped with COVID-19, namely, fatigue and shortness of breath. However, this character in the cancer vignette also suffered from a rash, loss of appetite, and a rapid weight loss—symptoms not associated with COVID-19. Furthermore, the vignette notes the appearance of these symptoms over a course of months. Therefore, it is significant that participants would include these symptoms in their mental models for COVID-19.

A closer analysis of the over-diagnoses may provide insight into how participants made sense of the material with which they were presented. For example, the first participant responded in the same way for the COVID-19 and cancer vignettes (thinking they were both COVID-19), citing mainly environmental and behavioral causes for disease causality and transmission (i.e., “contact with someone who was infected” and being close to someone who was ill). The pandemic messaging appears to influence their reasoning as they mentioned “face masks” and being “less than 6ft away” from an infected person.

The second participant was less confident in their overextension (“Maybe Covid?”) but responded to the subsequent questions using that same diagnosis. This participant gave different transmission explanations and time courses, despite suggesting the same diagnosis for both vignettes. The majority of their responses incorporate pandemic-specific language, such as “social distancing”, “mask wearing”, or referencing an infected person’s “droplets”.

The third and fourth participants followed a similar response pattern and, once again, suggested COVID-19 tentatively (“A virus, possibly Covid 19”; “Possible coronavirus”). They, too, used pandemic-specific language, suggesting “self-quarantine” or “isolation” and “social distancing” to prevent getting infected with COVID-19. The fourth participant specifically used near-verbatim responses for the questions regarding causality and transmission, suggesting their employed frameworks for the two vignettes were very similar, if not the same.

The final participant who diagnosed cancer as COVID-19 provided different causal explanations, once citing environmental factors (“contact with someone who was contagious” and the other time citing what the character did not do (“not washing hands and no social distancing”). The rest of their responses for the two vignettes were otherwise comparable.

Thus, participants’ mental models for COVID-19 appear broad enough to encompass symptoms of cancer, colds, and COVID-19 (conflating the diseases), even as they think more deeply about the vignettes by answering the illness-concept questions. While some participants were uncertain in their responses, each answered subsequent questions according to their diagnosis, despite being able to go back and edit their responses.

### 3.5. Beliefs about Cause, Transmission, Time Course, Prevention, and Treatment

Due to various forms of attrition and survey design (i.e., not requiring participants to complete each question before proceeding), not all participants completed the entire survey: some skipped questions, others skipped entire sections, and some quit early. As a result, many sets of data were incomplete. In addition, since the goal of the study was to compare individuals’ responses for different diseases, some participants were excluded from the vignette-questions analysis (participants had to “accurately” diagnose the disease [i.e., provide the response intended by the symptoms presented] for at least two vignettes. Beyond this, participants who failed to answer more than three questions for a vignette were excluded for that vignette [if they responded to enough questions for the other two illnesses, data were kept]. Full responses to all vignettes were ideal for comprehensive comparisons, but responses for at least two diseases still allowed for some comparison. Based on these criteria, 59 participants were excluded due to giving unintended illness diagnoses. Filtering out remaining insufficient responses, 33 responses for the cancer vignette remained, 61 for COVID-19, and 51 for the cold). Consequently, data from 63 participants remained. The following analyses refer to the subset of participants who both diagnosed the illness intended by the vignette and provided complete responses to at least two of the vignettes.

Of the remaining 63 participants, the majority were aged 55–64 (33.3%) and 18–24 (30.2%). Most participants identified as female (76.2%) and white (98.4%). The majority were highly educated, with 66.7% (*n* = 42) having completed a bachelor’s degree or above. The majority (66.7%) either did not respond (15.9%) or indicated having had no medical experience (50.8%). The most represented religious group was Catholic (42.9%), followed by Protestant (15.9%) or “spiritual, but not religious” (15.9%).

Chi-square goodness-of-fit tests showed that participants attributed different types of causes for COVID-19 and the cold, but not cancer. In the COVID-19 vignette, participants gave primarily biological agent or environment-based causal explanations, χ^2^(4, *n* = 59) = 35.49, *p* < 0.00001. Participants explained the cold as being caused by environmental factors χ^2^(4, *n* = 50) = 24.91, *p* = 0.00005. No clear pattern was observed for cancer, χ^2^(4, *n* = 23) = 7, *p* = 0.14. As seen in Table 1, the most frequent cause cited for COVID-19 is biological agent, while the most frequent cause cited for the cold is the environment. Cancer was explained mainly by a combination of factors.

The next question asked participants how the disease in question might be transmitted. Here, there were no significant patterns in any of the illness vignettes (cancer, χ^2^(3, *n* = 5) = 1.22, *p* = 0.75; COVID-19, χ^2^(3, *n* = 61) = 6.34, *p* = 0.096; cold, χ^2^(3, *n* = 50) = 3.44, *p* = 0.33). But response frequencies showed that participants tended to provide both environmental/behavioral and biological explanations (34.43%) for COVID-19, whereas cold transmission explanations referred to mainly environmental/behavioral factors (35.29%). Most participants agreed that cancer is not a contagious illness.

The fourth question regarded the time course of the given disease. Response patterns differed significantly across the three vignettes, as the majority of participants offered a shorter timeline for the two viruses (COVID-19, χ^2^(4, *n* = 58) = 84.54, *p* < 0.00001 and cold, χ^2^(4, N = 51) = 101.14, *p* < 0.00001) than for cancer (χ^2^(4, *n* = 27) = 40.5, *p* < 0.00001). The majority (78%) of participants said symptoms of a cold would likely arise within 1–7 days of contracting the illness, while responses for COVID-19 were more consistent with public-health guidelines at the time: 62.30% indicated symptoms could arise at any time up to 2 weeks. In contrast, over half of participants reported that cancer symptoms could take months or even a year to arise.

Participants were also asked how one might prevent themselves from getting the illness in question. Significantly more participants suggested precautions against biological and environmental agents for COVID-19 than other prevention routes (i.e., biological immunity), χ^2^(3, *n* = 61) = 117.59, *p* < 0.00001. This difference likely arises from the great deal of consensus in the COVID-19 vignette, as nearly 90% participants provided a biological/environmental precaution-based response. A similar pattern of biological/environmental-agent prevention measures appeared for the cold as well, χ^2^(3, *n* = 50) = 23.28, *p* = 0.00004. Here, though, participants showed less agreement and gave responses that fell into the “other” or both agent and immunity categories. A significant majority of cancer responses (45.45%) fell into the “other” category, χ^2^(3, *n* = 28) = 21.75, *p* = 0.00007.

The final question was aimed at participants’ beliefs about illness treatment. Participants provided significantly more biomedical responses for the cancer and COVID-19 vignettes than rest and nutrition or combination suggestions (cancer, χ^2^(3, *n* = 30) = 59.88, *p* < 0.00001; COVID-19, χ^2^(3, *n* = 57) = 12.68, *p* = 0.0054). Cancer more often requires biomedical intervention, as indicated by over 80% of participants. COVID-19 responses were more distributed, as a majority (36.07%) of participants suggested biomedical treatments, but others gave many “other” suggestions, perhaps indicative of the virus’s novelty not only to lay people but scientists and doctors as well. Cold treatment suggestions more commonly referred to resting and providing one’s body with proper nutrition and hydration, relying less on medicine and medical professionals, χ^2^(3, *n* = 47) = 8.75, *p* = 0.033.

## 4. Discussion

Participants relied on biological- and environmental-based reasoning in the domains of cause, transmission, and prevention for COVID-19 and colds but had a less cohesive understanding of cancer-related concepts. The results of our study show that, for most aspects of illness, participants who correctly identified the intended illness in the vignettes distinguished between colds, COVID-19, and cancer in their mental models.

In fact, participants used distinct mental models to answer the questions for COVID-19 and the common cold. Most COVID-19 responses fell into the biology-based categories while cold responses involved more environmental factors. This is a notable difference considering both diseases are viral and can be both transmitted and prevented in similar ways. Their more scientific understanding of COVID-19 may be a result of participants’ reliance on the most readily available framework for COVID-19, such as the one presented by authorities in research-based organizations like the CDC via public briefings and other news media. Though the common cold is spread in the same way as COVID-19, participants may lack the same CDC-supported biological association. Instead, participants may rely more on personal experience or common beliefs to form their mental models of colds. For example, some participants cited folk-biological explanations for catching a cold such as being cold and wet for too long—a common “cold weather” theory [17,18]. With this informal, more intuitive mindset, it is also possible for participants to base their assumptions of how they contracted a cold on contextual factors, overshadowing biological explanations.

That participants have separate mental models for somewhat similar diseases is critical to understanding how people reason about COVID-19. If most people’s understanding of COVID-19 is based in biology, then misconceptions should be combated with science. Science-based instruction aimed at correcting misbeliefs could use participants’ existing understanding of viruses as biological agents but redirect this knowledge to more accurate concepts, especially if lessons capitalize on causal reasoning (i.e., [19]). For example, at the onset of the pandemic, biological reasoning regarding germs and droplets led some individuals to disinfect their groceries. While data do not support this as a necessary measure [20], the concept of wearing masks to prevent inhalation of droplets and other airborne particles follows a similar (though science-based) line of reasoning. It is important to note that even biology-based understandings can reflect misinformation.

We find the cases of overextending COVID-19 diagnoses particularly interesting. It is remarkable that participants would more often include symptoms of cancer in their mental models for COVID-19 over symptoms for the cold, which are both less severe and more common. Some mainstream political discourse surrounding COVID-19 at the time of data collection downplayed the severity of the disease (i.e., [3]) which may have led individuals down the path of associating COVID-19 with the common cold. At the same time, scientists continued to discover more about COVID-19, including its potential for airborne transmission (i.e., [4]). Other research and instances of “long COVID” may also incite fear in individuals [21,22], altering their mental models for the illness. It seems plausible that the type of discourse with which an individual interacts may influence their mental model to either more extreme or more moderate conceptions of COVID-19.

It is possible that the participants whose mental models of COVID included symptoms of cancer represent more COVID-conscious individuals, who are well aware of the risks, and, perhaps, even are hypersensitive to COVID-19, hence their inclusion of anything remotely related to COVID-19 in their responses. This may be a product of their illness behavior, especially as it relates to COVID-19 specifically [23]. If participants are more concerned about COVID-19 and falling ill in general, they may be unwilling to miss anything related to the pandemic, a more conscious effort to react to anything that had even a chance of being COVID-19. This lower threshold would contribute to an over-extension of COVID diagnoses and, subsequently, influence a participant’s pattern of responses. In addition, constant updates about the course of the pandemic, daily death tolls, and other pandemic-related news dominated the media. This onslaught of COVID-19 information could also increase participants’ propensity to label anything slightly related to the new disease as COVID-19.

By varying the order in which participants received the vignettes, we set up the possibility for “priming” participants to think under the guise of COVID-19; this could have contributed to their increased sensitivity to think in pandemic-related ways. However, five of the seven participants received the vignette they misdiagnosed as COVID-19 first, eliminating the possibility of being primed by the COVID-19 vignette. Thus, if any sort of prime did take place, it was a result of conditions external to this study, such as the general societal discourse surrounding the pandemic.

Another explanation of participants’ over-extensions is that they, having been embedded in news around COVID-19 (whether they sought it out or not), have become “experts” of sorts. With regular updates and new information provided by press conferences from governmental agencies (such as the CDC and the FDA), the WHO, as well as social media, participants were barraged with COVID-19 information on a routine basis. Whatever information participants took away from these messages would have likely influenced their mental models, and, since COVID-19 news was often the top story of most days, participants may have been predisposed to labeling any illness that even slightly resembled the novel virus as COVID-19. Discourse rarely acknowledged other illnesses and the default was COVID-19. This is relevant as medical professionals who are specialized in their domain tend to be biased in their diagnoses, more frequently identifying participants as having ailments that fall within said domain [24].

A similar phenomenon may be occurring with the present participants, where the constant discourse surrounding the pandemic creates a feeling of “COVID-19 expertise” or saturation in individuals, making them more likely to diagnose the vignette characters as having COVID-19. Since their conceptions of illness was overwhelmed with information about COVID-19, it may have been the most readily available (and, seemingly, plausible) diagnosis. As shown in the over-extension diagnoses, participants were not always certain in their diagnoses, suggesting this phenomenon may be happening in a weaker way than with medical professionals. The majority of participants who over-extended COVID-19 diagnoses did not have medical experience, so their judgments were not based on true expertise.

There are some limitations present in this study. One concern is its small sample size, especially after participant responses were removed from the data due to attrition and incompletion. Many participants—none of whom received compensation for participating—did not fully complete the survey. Some participants skipped questions, whole vignettes, or failed to even submit the survey (responses were auto-collected as-is after a set period of inactivity). Second, because all the questions were open-ended, and the questions for each vignette were the same, it is possible participants got bored or were unwilling to type out lengthy answers, which would limit our insight into their understanding. Finally, collecting solely open-ended responses resulted in qualitatively rich and diverse data. As a result, some nuance to participants’ responses was undoubtedly missed by our coding scheme.

Future researchers might consider recruiting a large and more demographically diverse sample. For ease of coding, it may also be prudent to change the survey style from entirely open-ended responses to multiple-choice questions whose options correspond with distinct coding categories. Although this would eliminate some nuance from participants’ responses, this may make it easier for participants to respond and decrease the levels of attrition observed in this study. Further research must also be conducted to see how illness behavior interacts with specific pandemic-related behavior [25], especially as the pandemic continues to weigh on the world.

## 5. Conclusions

This study found that participants were best at accurately diagnosing COVID-19 compared to the common cold or cancer. However, when participants provided incorrect diagnoses, there was a small but nevertheless interesting trend of misdiagnosing the other illnesses as COVID-19. In addition, the data suggest that participants hold distinct mental models for each of the three illnesses. Specifically, they were more likely to explain COVID-19 using biology-based reasoning but an environmental rationale for the cold. It was difficult to discern trends for cancer given its ambiguity in its presentation, treatment, and outcomes.

Overall, this study begins to fill the gap in the knowledge of how participants think and reason about COVID-19 early in the pandemic. Though the pandemic continues globally even today in 2022, the data collected provide a window into how COVID-19 was initially understood in the United States, and how it might be incorporated into people’s thinking about other illnesses. This base framework may continue to inform individuals in the present. In addition, these findings can inform our understanding of how people incorporate public-health information about a new disease into their existing cognitive frameworks for illness.

## Figures and Tables

**Table 1:** Most common response category for each illness dimension by vignette.

	Cancer	COVID-19	Cold
**Cause**	24.2% Combination 18.2% Other 18.2% Biological	39.3% Biological 34.4% Environmental	43.1% Environment
**Transmission**	84.9% Non-contagious	34.3% Environmental and Behavioral/Biological 31.2% Biological	35.3% Environmental/Behavioral
**Time Course**	54.6% Months to a year	62.3% Up to 2 w	78.4% 1–7 d
**Prevention**	45.5% Other 36.4% Biological immunity	86.9% Biological/ environmental agent	52.9% Biological/environmental agent
**Treatment**	81.8% Biomedical	36.1% Biomedical 29.5% Other	35.3% Both biomedical and rest/nutrition 29.4% Rest/nutrition

## Data Availability

Data from this study are available upon request.

## Appendix A

Cancer Vignette:

*Julia is a 52-year-old woman who has an appointment with a medical professional, complaining of fatigue and shortness of breath. She reports worsening exercise tolerance over the past few months and difficulty breathing over the past several days. Prior to this, she exercised three to four times weekly, but now she finds it difficult to keep up with her friends and family’s daily activities. A physical examination led to the discovery of a rash on her chest and back and swollen lymph nodes on both sides of her neck. She reports a loss of appetite and frequent spells of nausea and vomiting. Her doctor is also concerned about her rapid, unintentional weight loss of 15 pounds. Julia is starting to worry, especially since she has been experiencing increasingly longer periods of chills and weakness.*

Cold Vignette:

*Max is a 12-year-old boy who has not been feeling well. Over the past few days he has had a runny nose, and a sore throat that has now become a cough. Although he had been going to school, he complains about feeling dull and drowsy. Max is starting to feel frustrated that he is still feeling unwell and hopes that he will start to feel better soon.*

## Appendix B

### Coding Scheme

**Table A1:** Q3 (Causation) “Why do you think (X) got sick? Are there any other reasons?”.

Code	Response Contained…
Biological agent	Germs/virus or catalyst (e.g., droplets, coughing, and sneezing)
Biological ulnerability	Genetics, weakened immune system, age/preexisting condition, “run down”, lack of sleep, diet, allergies, and poor hygiene
Environmental	Tick bite, carcinogen exposure, smoking, people, school, crowds, weather, not socially distancing, vague environmental reference, sick person, and contaminated surface
Combination	Combination of the above
Other	Other (e.g., global pandemic, spontaneous, and bad luck)

**Table A2:** Q4 (Transmission) “Can other people contract what (X) is suffering from? If so, how are they most likely to contract this condition?”.

Code	Response Contained…
Biological	Exposure to visible/invisible biological agent, droplets, and coughing
Environmental/ behavioral	Sharing drinks, ticks, kissing, lack of precautionary behaviors, contact with others, contact in general, and surfaces without agent
Biological and environmental/ behavioral	Combination of the above
Other	Other (e.g., alternative or supernatural)

**Table A3:** Q5 (Time course) “After they first contract the condition, how long do you think it would take for someone to feel symptoms?”.

Code	Response Contained…
0–24 h	Immediately, a day
1–7 d	Anything from 1–7 d, 1 w
Up to 2 w	8+ –14 d, includes 2-w responses, 1–2 w, a few weeks
Months–years	Greater than weeks
Other	Other (e.g., depends, asymptomatic)

**Table A4:** Q6 (Prevention) “What, if anything, could (X) have done to stop himself from getting this condition?”.

Code	Response Contained…
Biological/ environmental agent	Washing hands, wearing a mask, not touching face, social distance, avoiding carcinogens, avoid allergens, isolating/ quarantine, avoid crowded areas, and don’t play outside
Biological immunity	General health, seeing doctor regularly, being screened for diseases, hygiene, eating well, sleeping, exercise, vaccine, vitamins, precautions against environmental/chemical hazards
Agent and immunity	Combination of the above
Other	Other (e.g., doing good)

**Table A5:** Q7 (Treatment) “What, if anything, would make (X) feel better? Is there a cure for someone with this condition?”.

Code	Response Contained…
Biomedical	See medical professional, seek medical treatment, medication, medical procedure, drugs/prescriptions, ventilator, chemotherapy, surgery/operation, and oxygen monitoring
Rest/nutrition	Rest, nutrition, diet, hydration, vitamins, isolation, and hygiene
Both biomedical and rest/nutrition	Combination of the above
Other	Other (e.g., psychological help, emotional support)
